# Supplementary figures and images for: Twenty-Five Years of Propagation in Suspension Cell Culture Results in Substantial Alterations of the Arabidopsis Thaliana Genome
Source: Genes (Basel). 2019 Sep 2;10(9):671. doi: 10.3390/genes10090671 (PMC6770967; doi:10.3390/genes10090671)

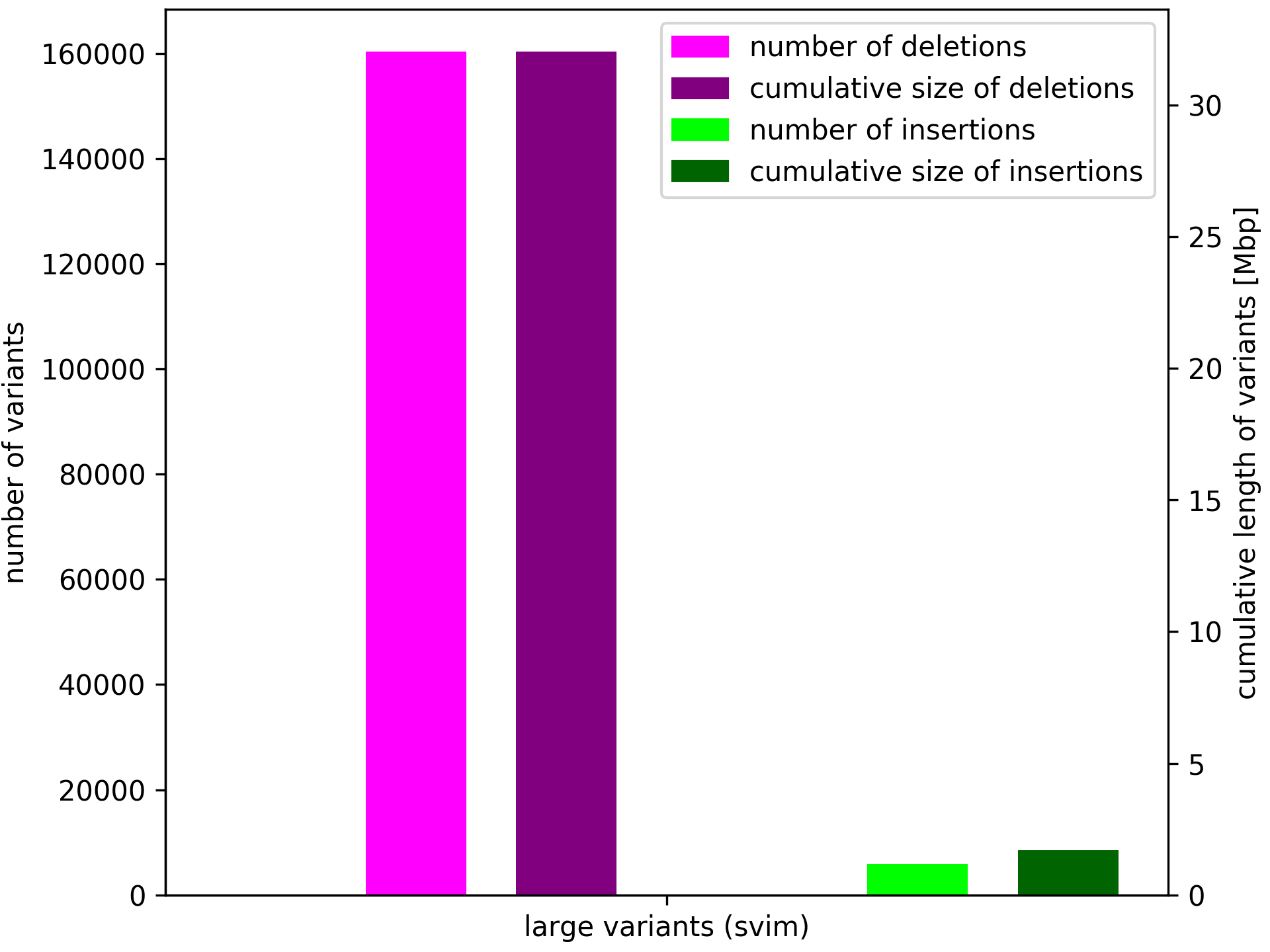

Supplement: Supplementary file 1 [file genes-10-00671-s001.zip › 20190813/Figure S1.png]

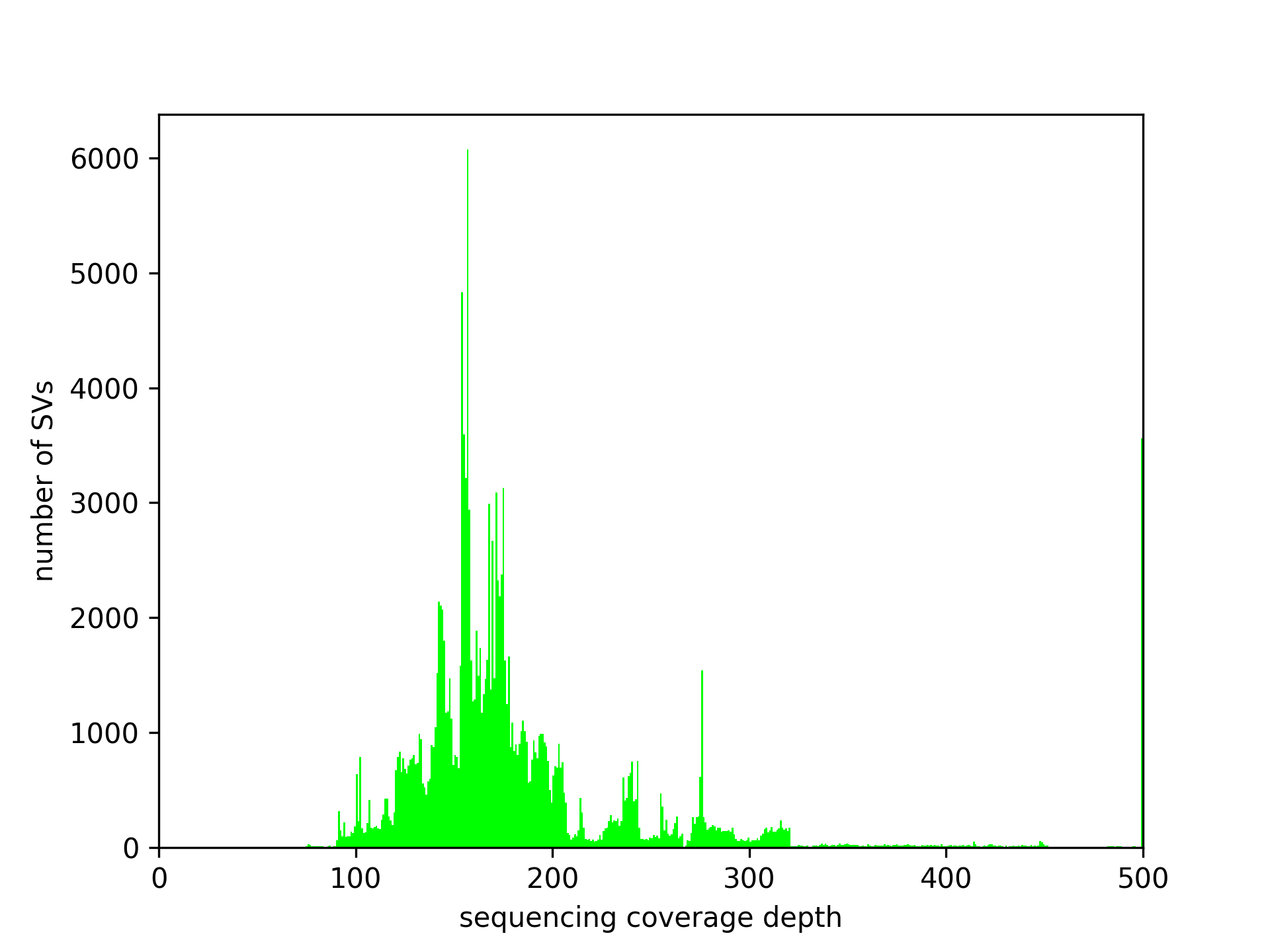

Supplement: Supplementary file 1 [file genes-10-00671-s001.zip › 20190813/Figure S2.png]

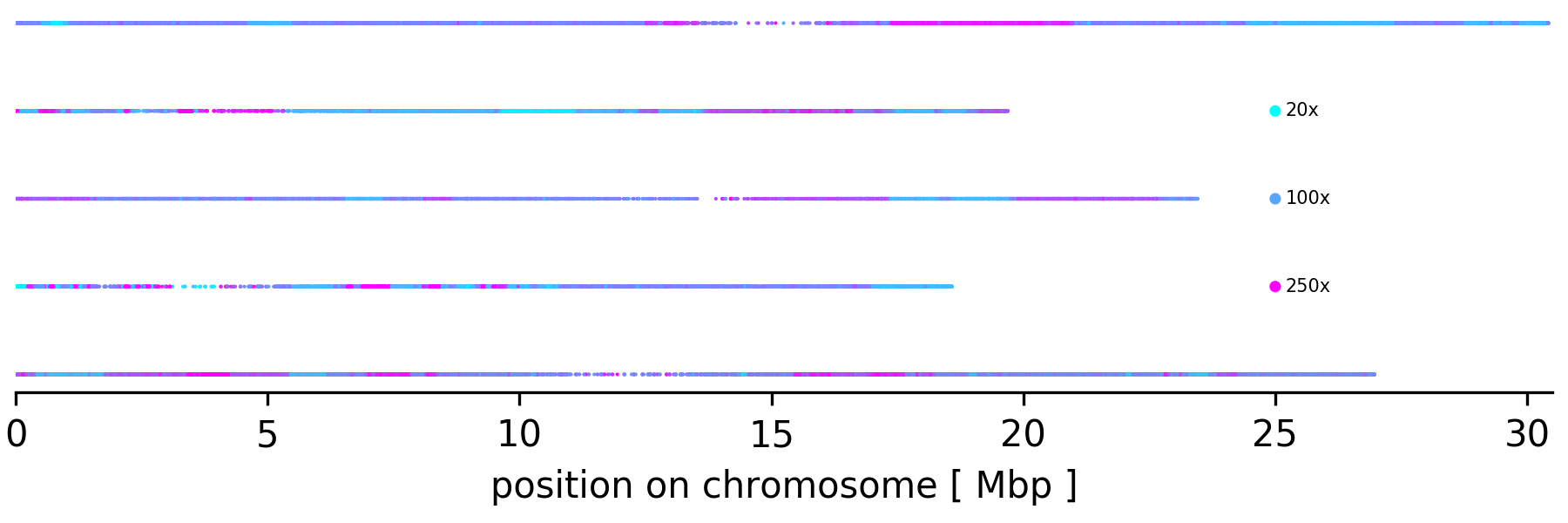

Supplement: Supplementary file 1 [file genes-10-00671-s001.zip › 20190813/Figure S4.png]

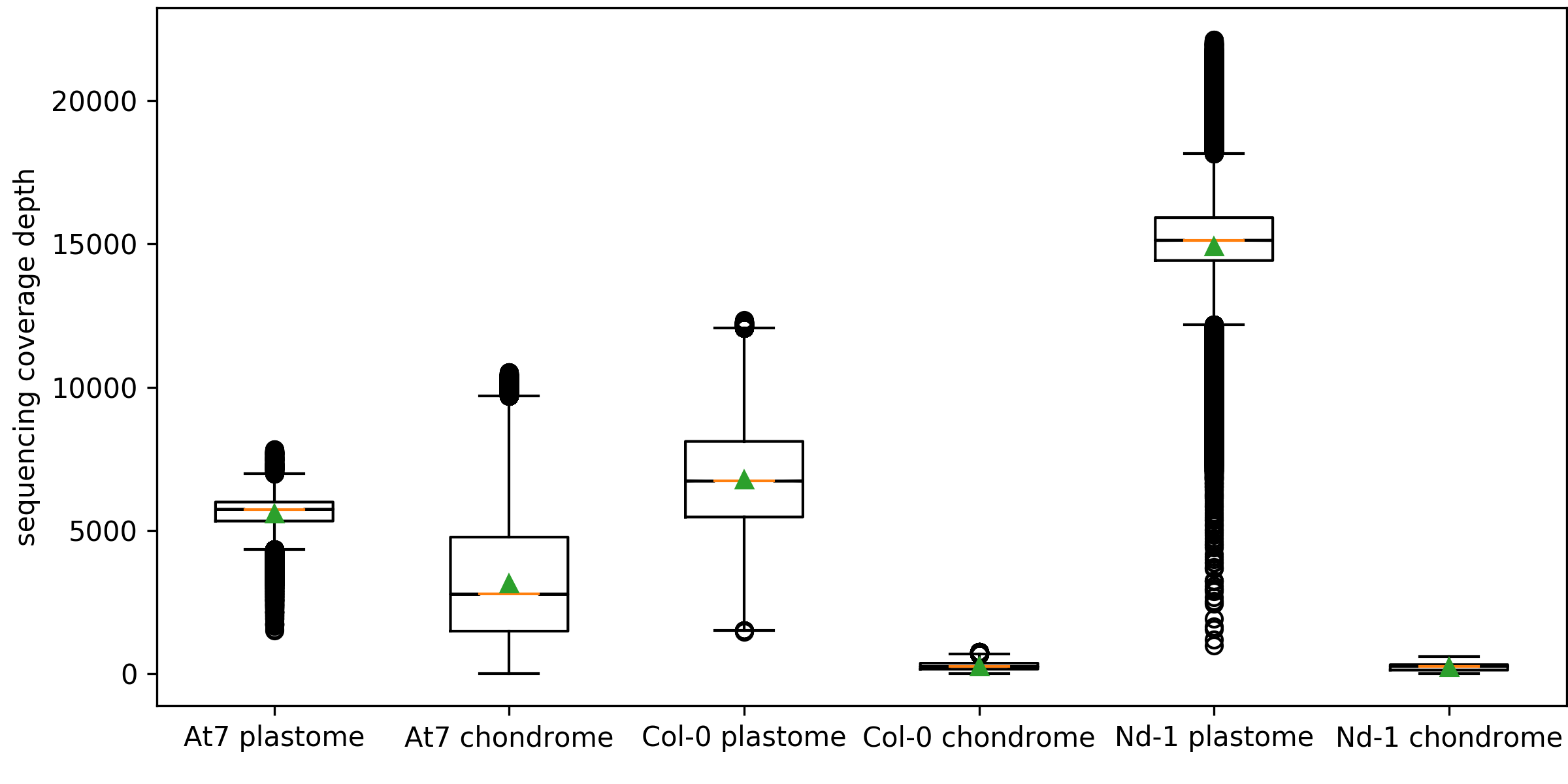

Supplement: Supplementary file 1 [file genes-10-00671-s001.zip › 20190813/Figure S3.png]

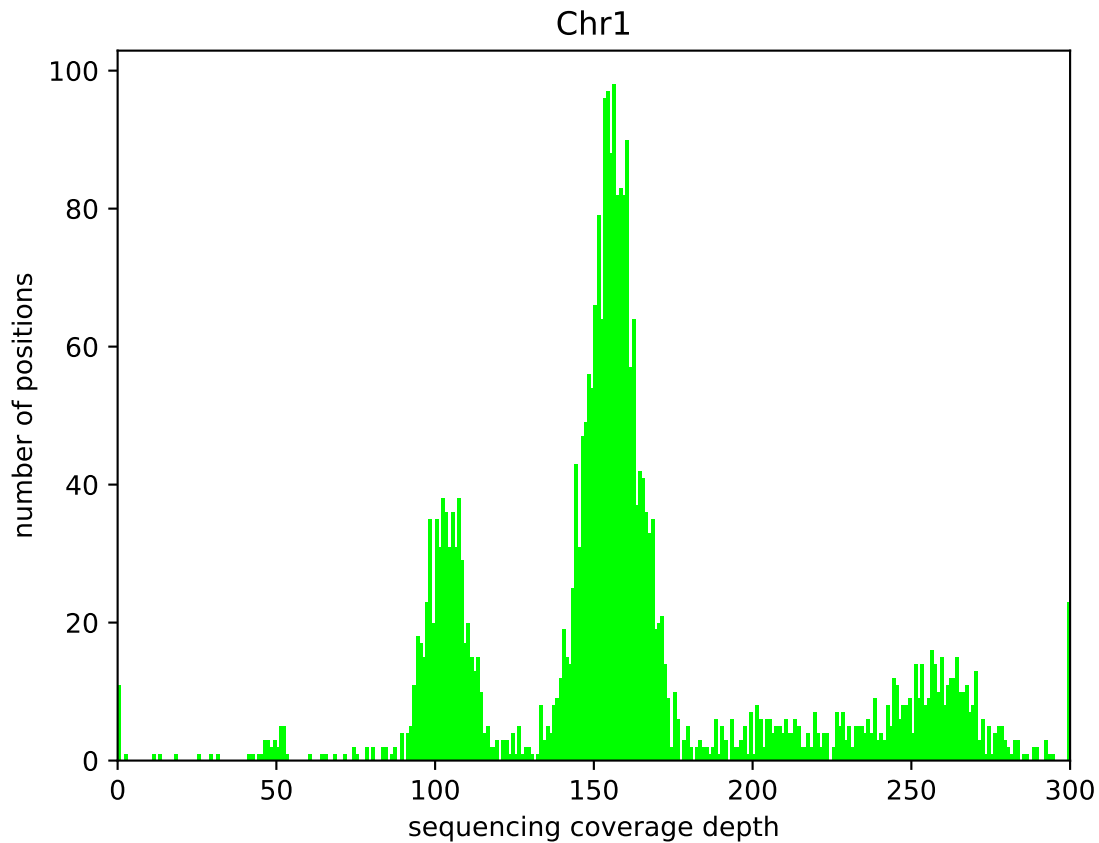

Chr2

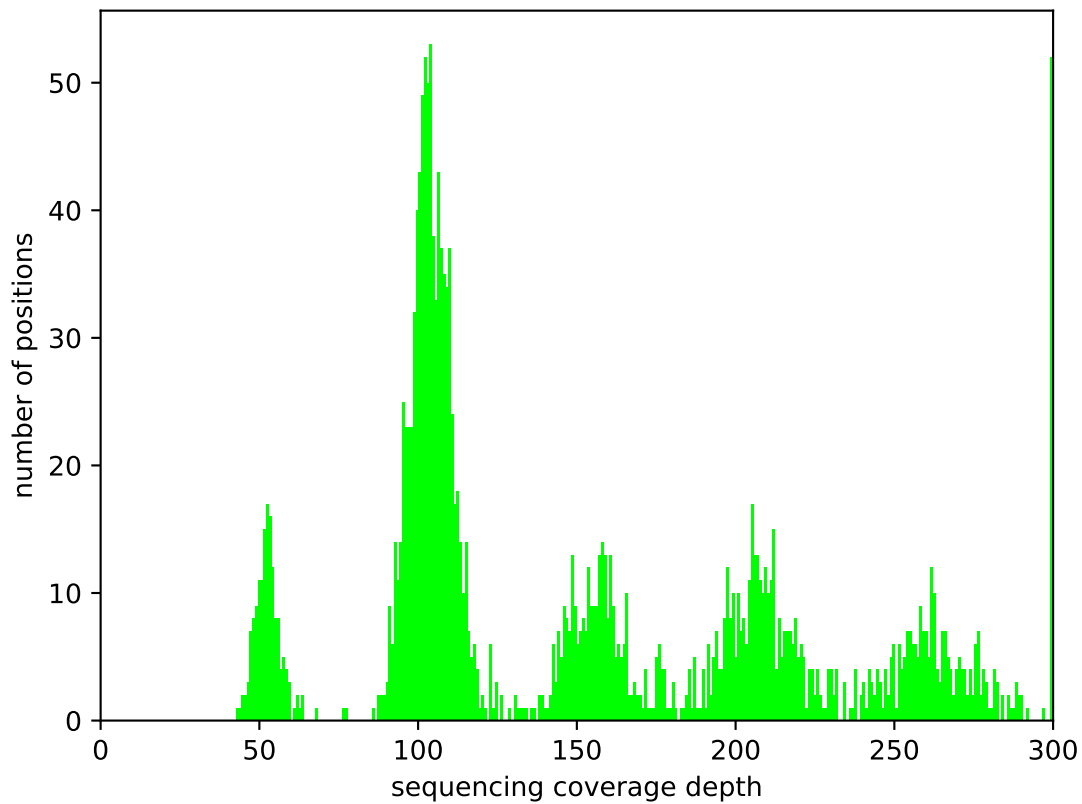

Chr3

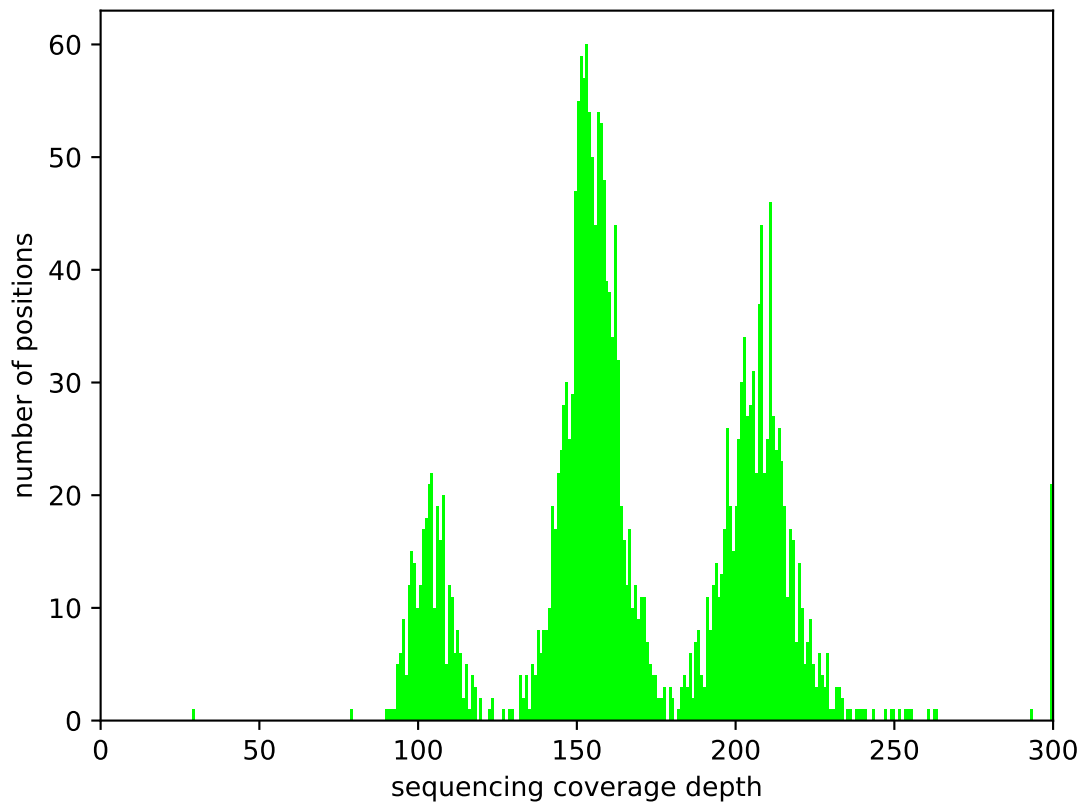

Chr4

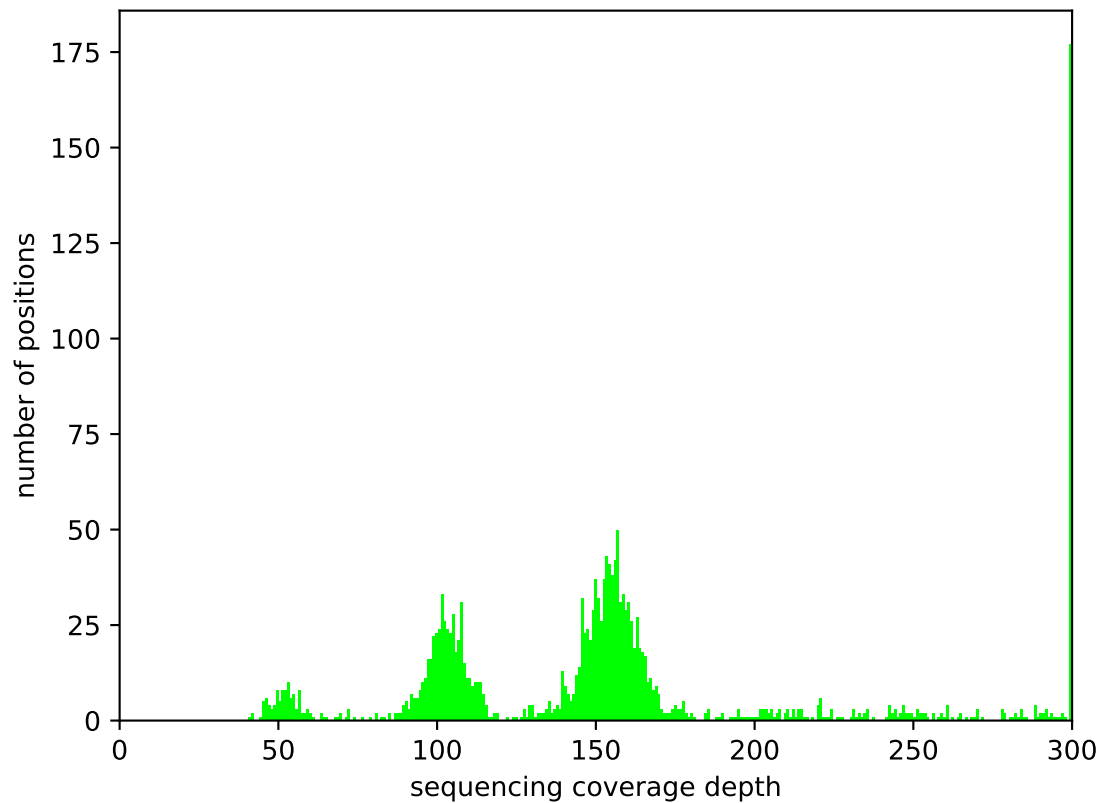

# Chr5

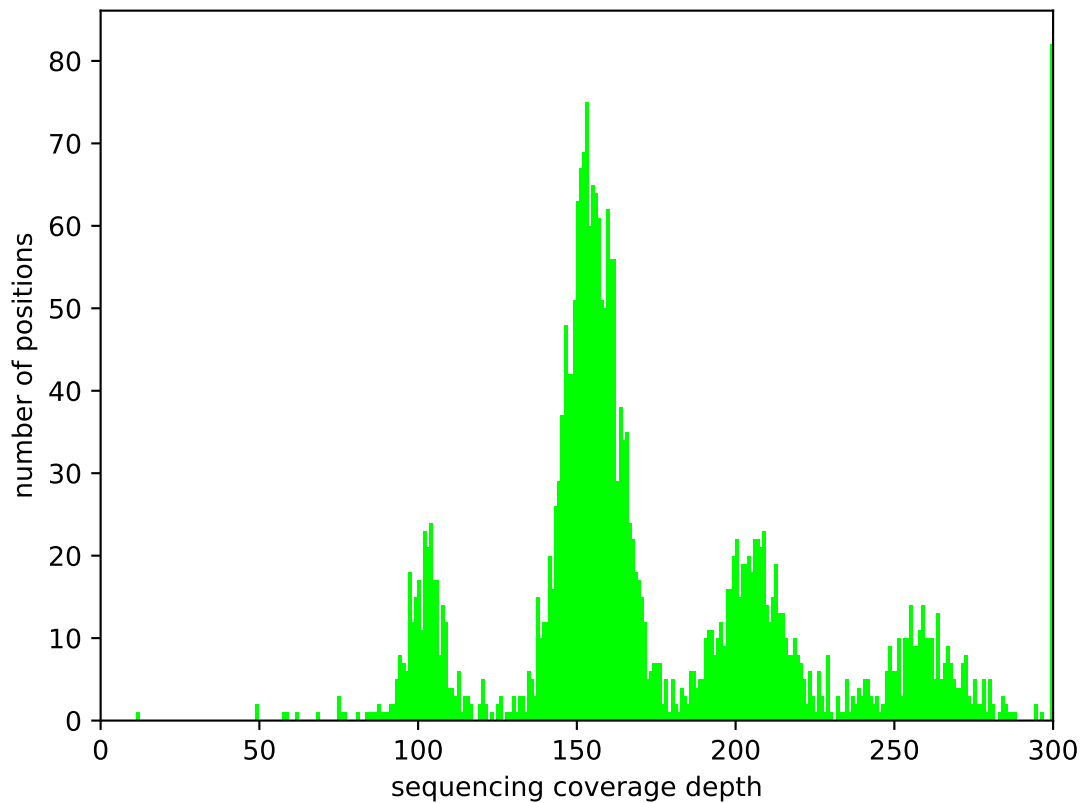

Supplement: Supplementary file 1 [file genes-10-00671-s001.zip › 20190813/File S1.pdf]
